# The complete mitochondrial genome sequence of the sakura shrimp, *Sergia lucens* (Crustacea, Decapoda, *Sergestidae*)

**DOI:** 10.1080/23802359.2018.1450671

**Published:** 2018-04-02

**Authors:** Satoshi Kawato, Reiko Nozaki, Hidehiro Kondo, Ikuo Hirono

**Affiliations:** Laboratory of Genome Science, Tokyo University of Marine Science and Technology, Tokyo, Japan

**Keywords:** *Sergestidae*, *Sergia*, sakura shrimp, mitochondrial genome, mitogenome

## Abstract

Here, we present the complete mitochondrial genome of the sakura shrimp, *Sergia lucens* (Crustacea, Decapoda, *Sergestidae*). The circular genome is 16,087 base pairs in length and contains 13 protein-coding genes, 22 tRNA genes, and two rRNA genes. The nucleotide composition of the *S. lucens* mitogenome is biased towards A + T (69.9%). The gene arrangement was identical to penaeid shrimps. Maximum likelihood phylogenetic analysis using the nucleotide sequences of 13 protein-coding genes placed *S. lucens* next to *Acetes chinensis*, supporting the conventional taxonomic relationship of *Sergia* and *Acetes*.

The sakura shrimp *Sergia lucens* is a small deep-sea crustacean that is primarily distributed to the waters off Japan and Taiwan (Imai et al. 2013). It has been an important fishery target in Suruga Bay, Japan. The genus *Sergia* belongs to the family *Sergestidae*, a group of pelagic shrimps that boast large biomass in temperate to tropical waters worldwide (Omori 1975). Despite the ecological and economic importance of *Sergestidae*, only one complete mitogenome (*Acetes chinensis*; GenBank accession no. NC_017600) has been reported. To overcome the deficit of sergestoid genetic information, we sequenced the whole mitogenome of *S. lucens*, one of the most economically important sergestoid shrimps.

A commercial supplier provided frozen *S. lucens* specimens that were sampled from a fishing ground in Suruga Bay, Japan. The exact geographic coordinate of the sampling site is unknown because the specimens were caught by trawling. We extracted genomic DNA from a whole shrimp body using the standard phenol–chloroform protocol. The DNA sample of the sequenced specimen is available from the authors. Intact specimens derived from the same sampling batch (MTUF-Ar 00016) are also available in the Marine Science Museum, Tokyo University of Marine Science and Technology. We constructed a paired-end DNA library using Nextera XT library preparation kit (Illumina, ‎San Diego, CA). We sequenced the library with the MiSeq platform (Illumina, ‎San Diego, CA) and obtained 5.8 million paired-end reads with a total of 720 million bases. *De novo* assembly of read data using CLC Genomics Workbench 6.5.1 (Qiagen, Germany) generated a putative mitogenome sequence, which we verified by Sanger sequencing. We used the MITOS pipeline (Bernt et al. 2013) for annotation and manually curated the MITOS output with penaeid mitogenomes. The complete mitogenome of *S. lucens* is available at DDBJ with accession no. LC368254. The raw Illumina read data can also be found in DDBJ Sequence Read Archive with accession no. DRR121359.

We conducted a maximum likelihood (ML) phylogenetic analysis of *S. lucens* and other shrimps using the 13 mitochondrial protein-coding genes. We used MAFFT version 7.310 (Katoh and Standley 2013) to generate multiple sequence alignments (MSAs) of the 13 protein sequences; the protein MSAs were then used as pgaligncodon 2.0 (Tanabe 2011) input to build codon-adjusted nucleotide MSAs. We excluded third codon positions of the protein coding genes and the rRNA sequences from the dataset because they exhibited GC content heterogeneity and may confound the analysis. We used Kakusan 4 (Tanabe 2011) to select the partitioning pattern (equal substitution rate among the genes) and the substitution model (GTRGAMMAX) used in RAxML 8.2.9 (Stamatakis 2006).

The complete mitogenome of *S. lucens* was circular and 16,087 bp in length. It contained 13 protein-coding genes, 22 tRNA genes, and two rRNA genes. The nucleotide composition was biased towards A + T rich (69.9%). The gene arrangement of the *S. lucens* mitogenome was identical to penaeid shrimps. The ML phylogenetic tree clustered *S. lucens* and *A. chinensis* in a single branch, albeit with a moderate bootstrapping support (77%), which conforms to the conventional taxonomic relationship of sergestoid shrimps (Figure 1).

**Figure 1.**
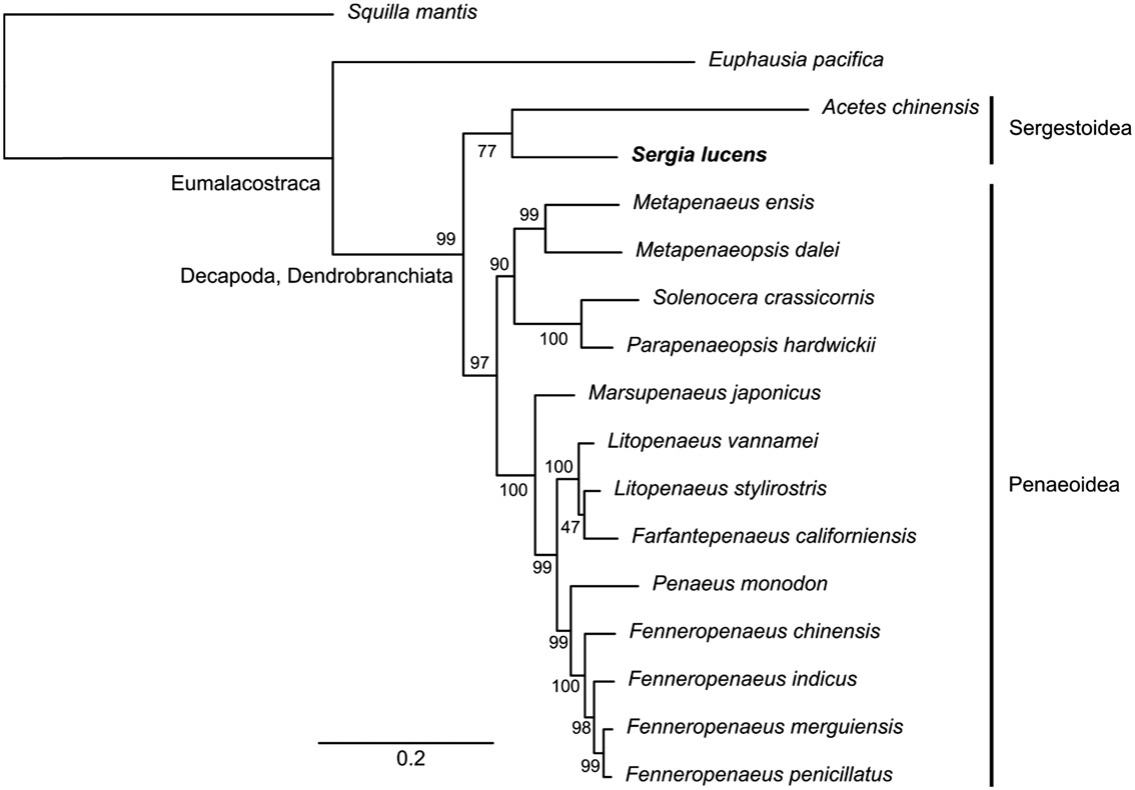
Maximum-likelihood phylogenetic tree of *Sergia lucens* and other 14 shrimps using two non-decapod crustaceans (*Squilla mantis* and *Euphausia pacifica*) as an outgroup. The concatenated nucleotide dataset of 13 mitochondrial protein-coding genes (excluding third codon positions) contained 11,040 sites. The bar below the tree indicates the number of nucleotide substitution per site, and the number beside the branches indicates the bootstrapping support (1000 trials) of each branch. The GenBank accession numbers of the mitogenome sequences used in the analysis are: *S. mantis* NC_006081; *E. pacifica* NC_016184; *Acetes chinensis* NC_017600; *Sergia lucens* LC368254; *Metapenaeus ensis* NC_026834; *Metapenaeopsis dalei* NC_029457; *Solenocera crassicornis* NC_030280; *Parapenaeopsis hardwickii* NC_030277; *Marsupenaeus japonicus* NC_007010; *Litopenaeus vannamei* NC_009626; *L. stylirostris* NC_012060; *Farfantepenaeus californiensis* NC_012738; *Penaeus monodon* NC_002184; *Fenneropenaeus chinensis* NC_009679; *F. indicus* NC_031366; *F. merguiensis* NC_026884; *F. penicillatus* NC_026885.
